# Formulation Approaches for Optimizing Omeprazole Stability in Oral Liquid Dosage Forms

**DOI:** 10.3390/pharmaceutics17050594

**Published:** 2025-05-01

**Authors:** Urszula Adamiak-Giera, Michał Gackowski, Damian Malinowski, Tomasz Osmałek, Marta Karaźniewicz-Łada, Anna Machoy-Mokrzyńska, Monika Białecka

**Affiliations:** 1 Department of Pharmacokinetics and Therapeutic Drug Monitoring, Pomeranian Medical University in Szczecin, 70-111 Szczecin, Poland; damian.malinowski@pum.edu.pl (D.M.); monika-bialecka@post.pl (M.B.); 2 Chair and Department of Pharmaceutical Technology, Poznan University of Medical Sciences, 60-806 Poznań, Poland; mgackowski4@gmail.com (M.G.); tosmalek@ump.edu.pl (T.O.); 3 Department of Physical Pharmacy and Pharmacokinetics, Poznan University of Medical Sciences, 60-806 Poznań, Poland; mkaraz@ump.edu.pl; 4 Department of Experimental and Clinical Pharmacology, Pomeranian Medical University in Szczecin, 70-111 Szczecin, Poland; anna.machoy.mokrzynska@pum.edu.pl

**Keywords:** omeprazole stability, pH-dependent degradation, immediate-release suspension, sodium bicarbonate, pharmaceutical compounding, gastrointestinal dissolution

## Abstract

**Background/Objectives:** This study aimed to evaluate the degradation of omeprazole suspension under various pH conditions and to propose recommendations for preparing compounded suspensions. Given the clinical need for alternative dosage forms for pediatric and geriatric patients and those with dysphagia, the research focused on assessing whether modifications in formulation composition—specifically the inclusion of sodium bicarbonate—could improve omeprazole stability, thus enhancing its bioavailability. **Methods:** Three formulations were prepared: O1, based on crushed enteric-coated pellets from a commercial product; O2, with crushed pellets suspended in an 8% sodium bicarbonate solution with glycerin; and O3, with pure omeprazole suspended in an 8% sodium bicarbonate solution with glycerin. Release studies were conducted using basket or paddle apparatus under conditions simulating fasted (pH 1.2 and 6.8) and fed (pH 6, 4.5, and 3) gastric and intestinal juices at 37 °C over 120 min. At predetermined intervals, samples were withdrawn and analyzed by a validated HPLC method with UV detection to quantify the released omeprazole. **Results:** The commercial enteric-coated product showed no release at a low pH, confirming its protective coating. In contrast, formulation exhibited significant degradation in acidic environments. The O2 formulation, benefiting from the buffering effect of sodium bicarbonate, showed improved stability compared to O1. Notably, formulation O3 yielded the highest drug recovery, with approximately 74% released at pH 6 and 65% at pH 6.8, demonstrating significantly better performance, as confirmed by statistical analysis (*p* < 0.05). **Conclusions:** The composition of omeprazole suspensions substantially influences the drug stability and release profiles. The O3 formulation, based on pure omeprazole with sodium bicarbonate, is recommended for immediate-release suspensions to enhance bioavailability. Further studies are needed to optimize conditions for pediatric use.

## 1. Introduction

Proton pump inhibitors (PPIs) are one of the most frequently used drugs, primarily in conditions associated with increased gastric acid secretion. The mechanism of action of PPIs is well described. All agents belonging to this group have weak base properties, allowing them to reach high concentrations in the acidic environment of the secretory canaliculi of the parietal cells in the gastric mucosa, where they inhibit the activity of one of the proton pump enzymes, H+/K+ ATPase, which transports hydrogen ions (H+) into the gastric lumen. The effect of this reaction is the reversible inhibition of hydrochloric acid secretion, affecting both basal and stimulated secretion, regardless of the stimulus triggering acid release [1,2].

The pharmacodynamic effect of PPIs depends on their pharmaceutical properties and pharmacokinetic parameters. The available oral forms of PPIs belong to the delayed-release category. For example, omeprazole (OME), lansoprazole, and esomeprazole are available in gelatin capsules containing enteric-coated granules (pellets), whereas pantoprazole and rabeprazole are administered in enteric-coated tablet form. This specialized pharmaceutical formulation, enteric coating, serves a dual role: protecting the PPI from degradation in the acidic gastric pH and delaying drug release until it reaches the absorption site—the intestine [3]. The efficacy of PPIs such as omeprazole depends on the proper course of pharmacokinetic processes. However, as demonstrated by studies conducted by Howden et al. [4], the enteric-coated formulation is poorly absorbed following a standard dose and exhibits significant interindividual variability concerning parameters such as the maximum plasma concentration (C_max_), the time to reach maximum concentration (t_max_), and the area under the plasma concentration–time curve (AUC).

The lack of sufficiently rapid relief of symptoms may lead to dose escalation or treatment discontinuation by the patient. Additional challenges in PPI therapy, including omeprazole, may arise in special patient groups, such as children over one year of age and unconscious patients hospitalized in intensive care units, where the need for a modified drug form (e.g., crushing) may unpredictably affect its pharmacokinetic parameters. A potential solution to these issues could be an immediate-release (IR) formulation, ensuring faster absorption and, consequently, a quicker onset of antisecretory action [5].

In the United States, the first oral immediate-release omeprazole preparation, Zegerid^®^ (manufactured for Santarus, Inc. by Patheon Inc., Whitby, Ontario L1N 5Z5, Canada), has been approved in the form of a powder for oral suspension. This medication contains omeprazole at doses of 20 mg or 40 mg, combined with 1680 mg of sodium bicarbonate. A pharmacokinetic comparison between immediate-release omeprazole and delayed-release formulations demonstrated that the IR formulation achieved a significantly shorter time to peak plasma concentration without notable differences in the AUC between the two forms. The absorption of omeprazole, as well as the onset of antisecretory action, is faster with the IR formulation. This is likely due to the rapid increase in the gastric pH following ingestion of omeprazole in a sodium bicarbonate suspension. Feurle et al. [6] demonstrated that sodium bicarbonate solution raises circulating gastrin levels within 30 min of oral administration. The rise in the circulating gastrin concentration may stimulate the parietal cells and promote the recruitment of H+/K+ ATPase molecules into the secretory canaliculi. The peak plasma concentration of omeprazole following administration of the IR formulation was observed after approximately 30 min, allowing rapid drug absorption by activated parietal cells and potentially leading to the irreversible inhibition of a substantial portion of available H+/K+ ATPase molecules.

Unlike delayed-release PPIs, immediate-release omeprazole contains sodium bicarbonate, which replaces the enteric coating to protect the active substance from degradation by gastric acid. In Poland, ready-to-use immediate-release omeprazole preparations are not available. However, a pharmaceutical raw material for prescription compounding has recently been registered, enabling the preparation of an omeprazole oral suspension in an 8% sodium bicarbonate solution. The introduction of this new pharmaceutical raw material, omeprazole in powder form, significantly facilitates the preparation of pediatric suspensions and allows the formulation of the drug without mechanically damaging the capsule or releasing the pellets in patients requiring enteral feeding.

Omeprazole suspension with sodium bicarbonate may become an alternative for many patients; however, the literature on the potential variability of the pharmacokinetic transformations of this drug remains limited. These observations prompted the authors of this study to evaluate the degradation rate of omeprazole as a function of environmental pH and to propose recommendations for the compounding and administration of prescription omeprazole suspensions, which may help address some therapeutic challenges associated with this drug, particularly in the pediatric population and adults with dysphagia.

The aim of our study was to determine the degree of omeprazole degradation depending on the pH of the dissolution medium as well as the composition and method of preparation of the suspension. Furthermore, the study aimed to assess the effect of sodium bicarbonate on the degradation of omeprazole and to determine whether the use of pharmaceutical-grade raw materials, in comparison to crushed pellets, significantly influences the degradation rate of the active substance.

To the best of our knowledge, this is the first study to directly compare the extemporaneously prepared omeprazole suspensions that are commonly used in clinical settings in Poland. By simulating biorelevant gastrointestinal conditions, we aimed to provide evidence-based recommendations for pharmacy-compounded immediate-release formulations. This approach addresses a practical gap in pharmaceutical care for populations requiring alternative dosage forms, particularly in the absence of commercially available immediate-release omeprazole products in many European countries.

## 2. Materials and Methods

Materials: Omeprazole, glycerin, and sodium bicarbonate were purchased from Fagron (Kraków, Poland). Phosphate release buffer (pH 6.8) and hydrochloric acid-release buffer (pH 1.2) were obtained from Pol-Aura (Morąg, Poland). FEDGAS (early, mid, late) biorelevant dissolution media simulating gastric fluids after food, including GEL and buffers (pH 6, 4.5, and 3), were purchased from Biorelevant (London, UK). Potassium dihydrogen phosphate, triethylamine, and acetonitrile were from Merck (Darmstadt, Germany). All the reagents were of analytical grade, except for omeprazole, glycerin, and sodium bicarbonate, which were of pharmaceutically appropriate quality for preparation in pharmacy compounding conditions.

The commercial formulation of OME contained the following excipients: sucrose granules, hypromellose, povidone K25, sodium lauryl sulfate, heavy magnesium oxide, talc, methacrylic acid and ethyl acrylate copolymer 1:1 (30% dispersion), triethyl citrate, and magnesium stearate.

Preparation of the formulations: Four formulations (three self-prepared and one commercially available drug) were tested. The commercially available product consisted of capsules containing enteric-coated pellets. Formulation O1 was prepared by crushing the pellets from a commercially available drug using a mortar and pestle. The crushed pellets were subsequently placed in a release container. Formulation O2 was prepared by crushing the pellets from a commercially available drug using a mortar and pestle. The crushed pellets were suspended in an 8% sodium bicarbonate solution. Initially, a suspension concentrate was prepared by adding a portion of solution, and then the remaining solution was gradually incorporated. Finally, glycerin was added and mixed thoroughly with the rest of the suspension. Formulation O3 was prepared by suspending pure omeprazole in an 8% sodium bicarbonate solution. Initially, a suspension concentrate was formed by adding a portion of solution and gradually adding the remaining solution. Finally, glycerin was incorporated and mixed with the suspension. The composition of the self-prepared omeprazole suspensions is presented in Table 1.

In this study, the formulations were prepared using a different approach from that previously described in the literature [7,8]. This decision was based on the fact that the pH of glycerol (85–100 g/L) is 5. Given that omeprazole is unstable at pH levels below 7.4, glycerol was added at the final stage of the drug preparation process [7]. The tested formulations, O1 to O3, were selected based on clinical practice, as these three compositions are the most commonly used in hospital and community pharmacies as well as in pediatric and geriatric wards. It should also be noted that the composition of suspension O3 is recommended in the literature [8]. According to the literature, the stability of suspension O3 is reported to be 14 days when stored at 2–8 °C [8].

The pH measurement was carried out to determine the acidity or alkalinity of the formulation. The analysis was performed using a calibrated pH meter (Hanna Instruments HI221, Olsztyn, Poland) at room temperature. Before each measurement, the electrode was rinsed with distilled water and gently dried with a lint-free tissue to avoid cross-contamination. All measurements were conducted in triplicate, and the results are presented as mean ± standard deviation.

Release studies: The release study of omeprazole from commercially available capsules containing enteric-coated pellets was conducted using a rotating basket apparatus, whereas self-prepared omeprazole suspensions (O1, O2, O3) were tested using a rotating paddle apparatus (Agilent 708-DS, 850-DS; Santa Clara, CA, USA). The test media included fasted-state buffers with pH 1.2 and 6.8 as well as fed-state buffers mimicking stomach contents after a meal, reflecting the early (pH = 6), mid (pH = 4.5), and late (pH = 3) stages of gastric emptying. The test was performed in 500 mL of medium for fasted-state conditions and 250 mL for fed-state conditions. Each experiment was conducted in triplicate (*n* = 3) at 37 °C for 120 min. The rotation speed of the baskets and paddles was set to 75 rpm [9]. The study involved placing the content of one capsule (omeprazole 20 mg) or 10 mL of suspension into each of three baskets/beakers (*n* = 3). During the test, 1 mL samples were withdrawn from each beaker at predetermined time points: 1, 5, 10, 20, 60, 90, and 120 min. After each sampling, the chambers were replenished with an equivalent volume of fresh buffer. The amount of released omeprazole was quantified using a validated HPLC method with UV detection. The results are presented as cumulative release profiles.

HPLC analysis: The omeprazole concentration in the buffers was determined by a validated high-performance liquid chromatography method with ultraviolet detection [10]. The chromatographic system (HPLC, Agilent, Santa Clara, CA, USA) consisted of an HP 1100 pump, an HP 1100 autosampler, and an HP 1100 ultraviolet detector. The isocratic mobile phase was composed of a buffer solution consisting of 25 mM potassium phosphate monobasic in water containing 0.25% triethylamine and acetonitrile (350:650 *v*/*v*). The pH of the mobile phase was adjusted to 6.5 with 85% phosphoric acid. The flow rate of the mobile phase was isocratic at 1 mL/min at a temperature of 22 °C. The wavelength of UV detection was set at 290 nm. Chromatographic separations were achieved in the Supelcosil^TM^ LC-8-DB column (5 μm, 15 × 4.6 mm) (Merck, Darmstadt, Germany). The lower limit of detection was 0.05 µg/mL, and the lower limit of quantitation for omeprazole was 1 µg/mL.

### Statistical Analysis

The distribution of data did not differ significantly from the normal distribution (Shapiro–Wilk test), so they were compared between groups using the Student’s *t*-test. Statistical analysis and dissolution profile comparisons were performed using STATISTICA PL, ver. 13.3 software (StatSoft, Inc., Tulsa, OK, USA). *p* < 0.05 was considered statistically significant. A comparison between the O1, O2, and O3 formulations was performed at the 30 min and 120 min time points.

## 3. Results

The pH values of formulations O2 and O3 were measured to assess their physicochemical properties. Each formulation was analyzed in triplicate, and the results are expressed as mean ± standard deviation (SD). The obtained values are presented in Table 2.

**Figure 1:**
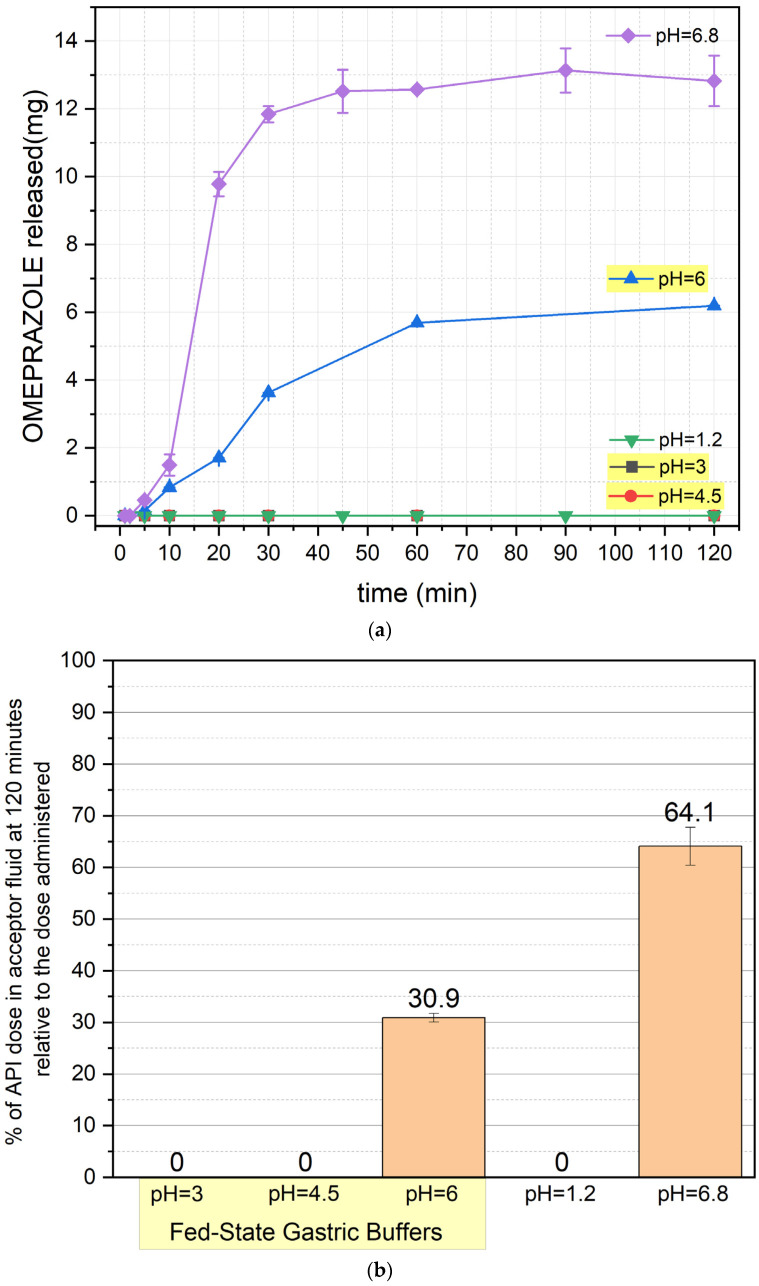
(**a**) OME release profile from a commercially available drug (mean value ± SD from *n* = 3). (**b**) Percentage of OME left after 120 min of the test (mean value ± SD from *n* = 3) for the commercially available drug. The yellow background of the pH values indicates the results with fed-state buffers. % of API (active pharmaceutical ingredient)—% of omeprazole.

**Figure 2:**
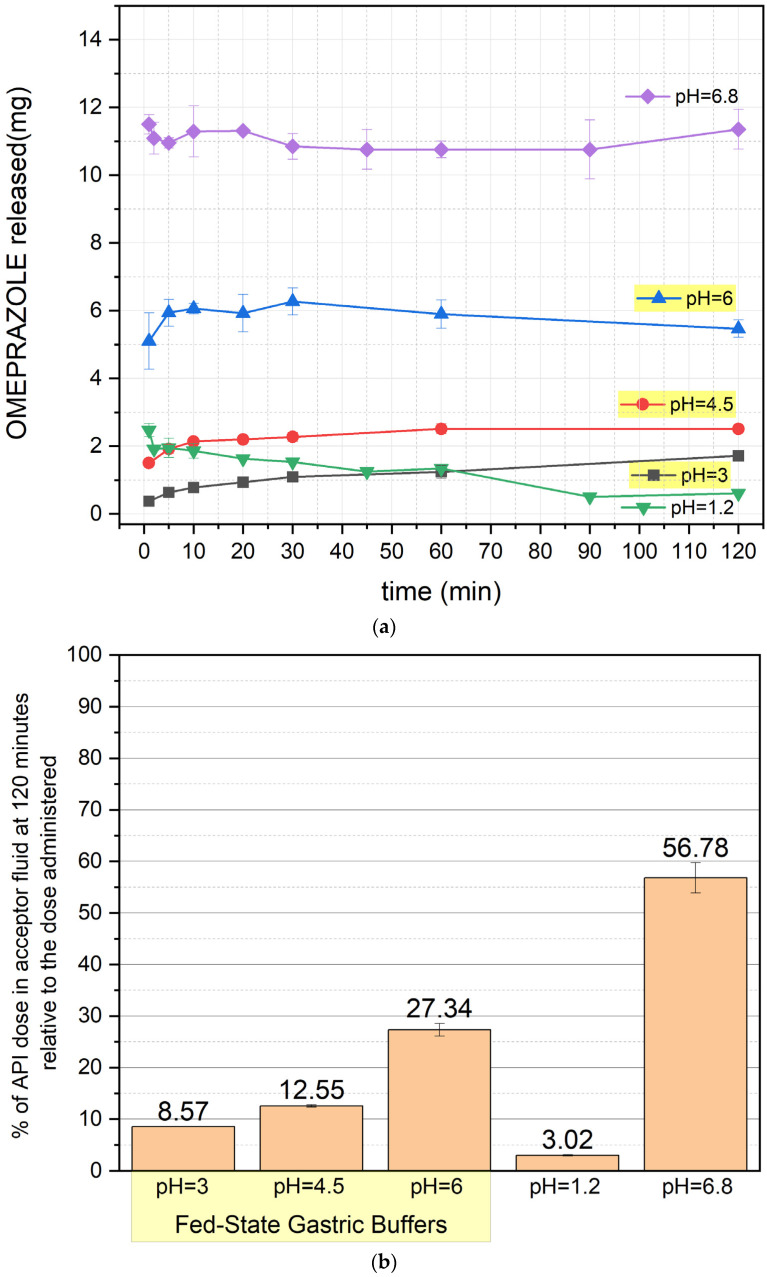
(**a**) OME release profile of formulation O1 (mean value ± SD from *n* = 3). (**b**) Percentage of OME left after 120 min of the test (mean value ± SD from *n* = 3) for formulation O1. The yellow background of the pH values indicates the results with fed-state buffers. % of API—% of omeprazole.

**Figure 3:**
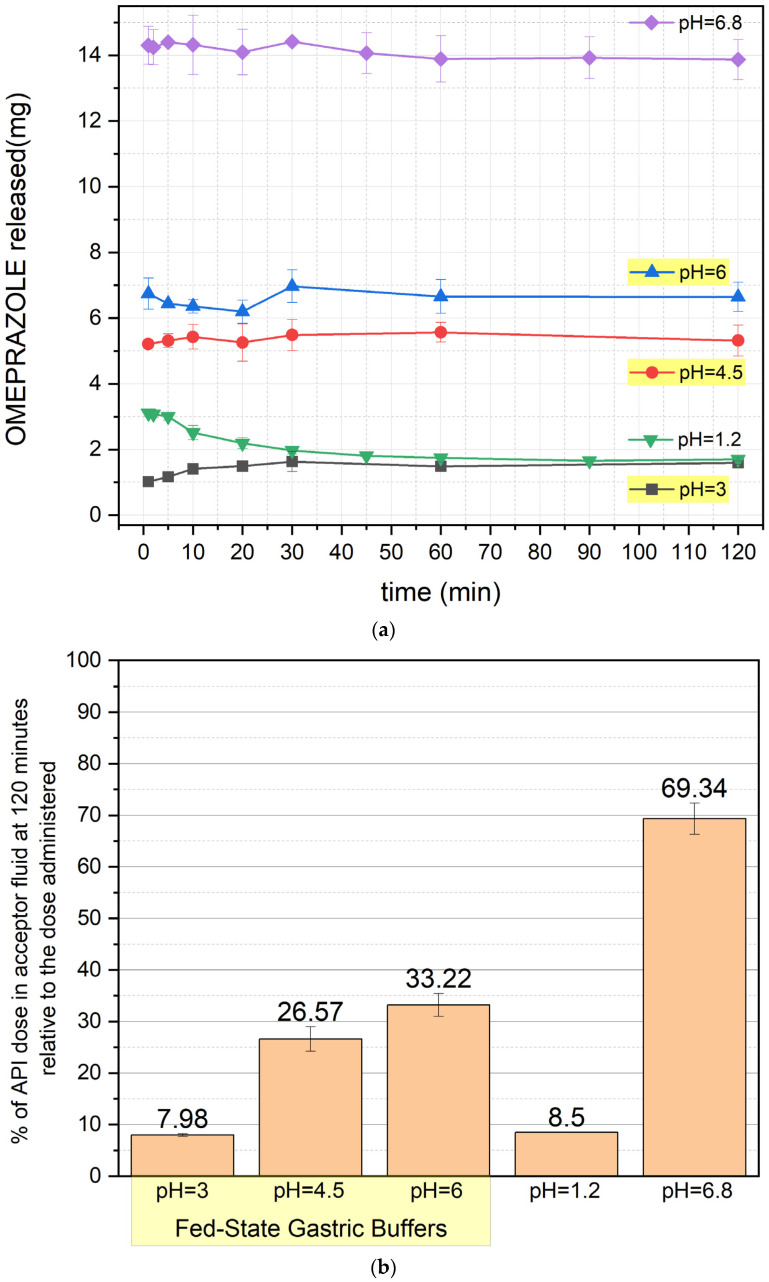
(**a**) OME release profile of formulation O2 (mean value ± SD from *n* = 3). (**b**) Percentage of OME left after 120 min of the test (mean value ± SD from *n* = 3) for formulation O2. The yellow background of the pH values indicates the results with fed-state buffers. % of API—% of omeprazole.

**Figure 4:**
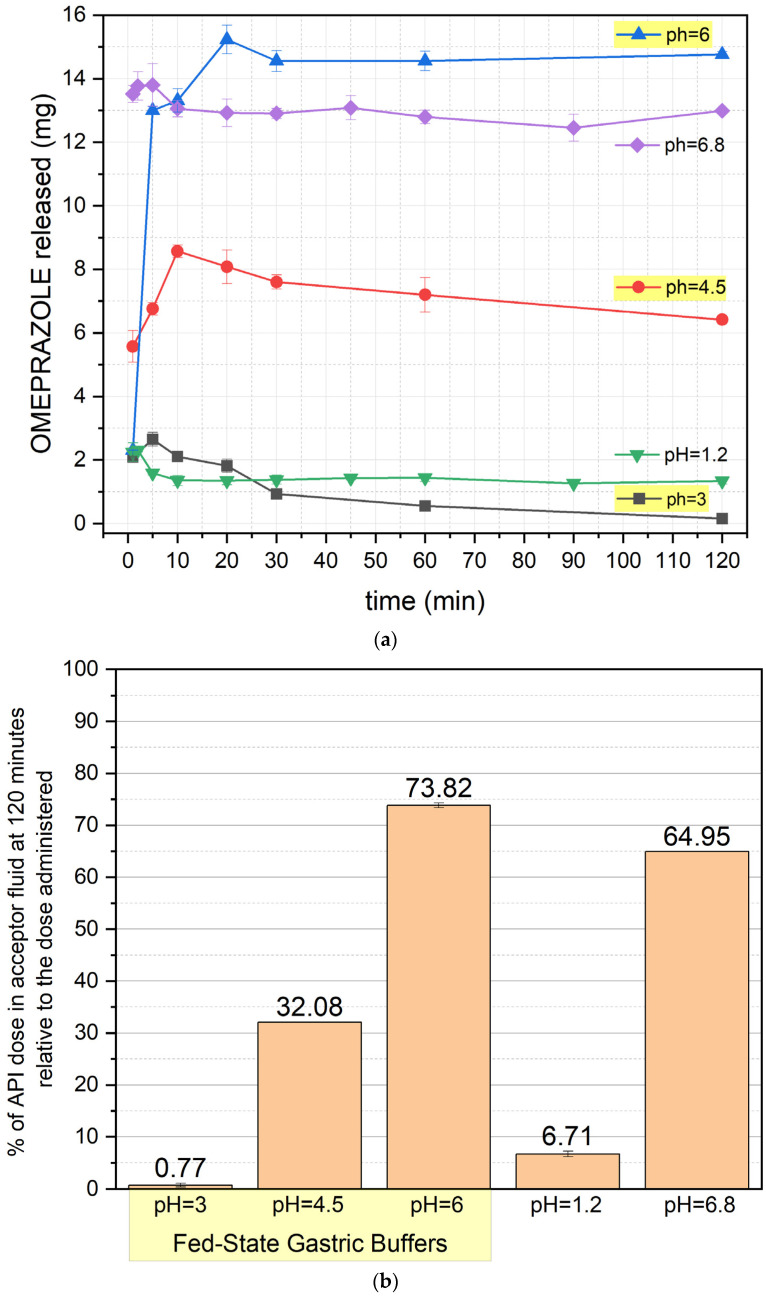
(**a**) OME release profile of formulation O3 (mean value ± SD from *n* = 3). (**b**) Percentage of OME left after 120 min of the test (mean value ± SD from *n* = 3) for formulation O3. The yellow background of the pH values indicates the results with fed-state buffers. % of API—% of omeprazole.

The results of the release study are presented in Figure 1a,b, Figure 2a,b, Figure 3a,b and Figure 4a,b (for the pellets and formulations O1, O2, and O3, respectively) (Supplementary Tables S1 and S2).

Figure 1 presents the release profile of OME from the commercially available formulation containing enteric-coated pellets. No release was observed under acidic conditions (pH 1.2, 3, and 4.5), confirming the protective function of the enteric coating, which prevents drug degradation from the first minute of the test. Release begins at pH 6 and 6.8, with distinct behaviors. At pH 6, the release occurs gradually over the first hour, after which it reaches a plateau. At pH 6.8, drug release is significantly faster, with most of the OME being liberated within the first hour, followed by stabilization. After 120 min, 30.9% of the dose is released at pH 6, while at pH 6.8, this value reaches 64.1%.

It is important to note that the enteric-coated formulation begins to release the drug at pH 6, which can be reached in the stomach after a meal and is also observed in the duodenum [11]. Considering the stability of omeprazole, which is unstable at pH levels below 7.4, it should be noted that a portion of the released dose is already exposed to degradation in the stomach or duodenum, where the pH increases to higher values.

Figure 2 illustrates the release profile of OME for formulation O1. At pH 1.2 and 3, the drug concentration is minimal, which confirms the noticeable degradation from the first minute of the experiment. At pH 4.5, release increases slightly but remains limited. After 120 min, 27.34% of the dose is released at pH 6, whereas at pH 6.8, this value reaches 56.78%. Release profiles are stable throughout the experiment.

The results indicate that if there is a need to administer the drug in the O1 formulation (which is strongly not recommended considering the results obtained for the other formulations), it should be given after a meal when the gastric content pH is at its highest.

Figure 3 shows the release profile of formulation O2, demonstrating improved OME stability compared to O1. At pH 1.2 and 3, release remains minimal, with degradation occurring from the first minute of the test. At pH 4.5, release increases slightly, though degradation remains evident. After 120 min, approximately 33% of the dose is released at pH 6; at pH 6.8, this value increases to around 70%. Release profiles are stable throughout the experiment.

A statistically significant difference (*p* < 0.05) was found when comparing O1 and O2, particularly at pH 1.2 and pH 4.5 (30 min and 120 min time points), indicating the stabilizing effect of NaHCO_3_ on omeprazole release. Moreover, the addition of sodium bicarbonate to the formulation stabilizes the drug itself, as the pH of the prepared omeprazole suspension reaches approximately 8.8, thereby reducing the pH-dependent degradation of omeprazole within the formulation [8].

Figure 4 presents the release profile of formulation O3, exhibiting significant differences from the previous formulations. At pH 1.2 and 3, drug release is minimal, with degradation visible from the first minute of the test. At pH 4.5, release increases moderately but remains restricted due to OME degradation. At pH 6, drug release is rapid and efficient, with a plateau reached after approximately 60 min. Under fed-state conditions (pH 6, 4.5, and 3), an initial increase in release is observed, followed by stabilization or a slight decrease. After 120 min, the highest drug recovery was recorded at pH 6 (~74%) and pH 6.8 (~65%), while at pH 4.5, this value was approximately 32%.

A distinct release pattern can be observed when analyzing the release profiles of the active pharmaceutical ingredient (API) from the O3 formulation. The initial increase in API concentration during the early phase of the test suggests that the API used (pure omeprazole) exhibits different physicochemical properties from the omeprazole contained in the finished pharmaceutical product. The pure API shows a slower dissolution rate than the drug substance in pellets.

This phenomenon may arise from the differing physicochemical properties of omeprazole found in the finished commercial product and the pure omeprazole in the formulation of O3. In particular, the presence of a different polymorphic form of omeprazole in the commercial products (omeprazole exhibits five polymorphic forms [12]) or variations in the particle size of the drug substance could contribute to these differences in dissolution behavior.

Additionally, variations in tₘₐₓ depending on the dissolution medium should be noted. The Cₘₐₓ was reached fastest in the postprandial fluid at pH 3 (5 min), followed by pH 4.5 (10 min) and pH 6 (20 min). This can be explained by the decreasing concentration of hydrogen cations (protons) in the formulation. The lower the pH is, the higher the number of protons present. Since the fluid simulates gastric juice after a meal, it also contains macromolecules that hinder interactions between the API and the protons. When the proton concentration is high, the probability of interactions between H⁺ and the API increases, leading to its degradation. As the proton concentration decreases, the likelihood of these interactions is reduced, allowing a prolonged dissolution phase before reaching the plateau phase.

A statistically significant difference was also observed when comparing O3 and O2 at all pH values (except for the alkaline pH 6.8), where the O3 formulation achieved significantly higher drug concentrations in the dissolution medium. This suggests a potential role for the excipients used to achieve enteric coating in the commercial product from which the O2 formulation was prepared, as they appear to influence the extent of API degradation.

**Figure 5:**
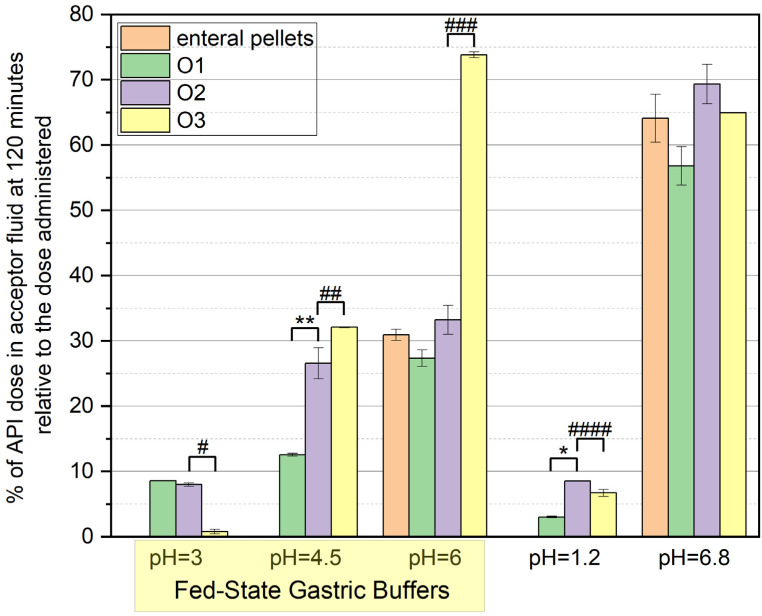
Bar graph showing the cumulative percentage of OME remaining in the dissolution medium at the 120th minute of the test. % of API—% of omeprazole (* *p* = 0.00001; ** *p* = 0.047; # *p* = 0.001; ## *p* = 0.036; ### *p* = 0.001; #### *p* = 0.02).

Figure 5 presents the cumulative percentage of OME remaining in the dissolution medium at the 120th minute of the test, comparing the release profiles of formulations O1, O2, and O3 (Supplementary Tables S1 and S2). A significant difference in drug release was observed between these formulations.

As previously stated, a statistically significant difference (*p* < 0.05) was found when comparing O1 and O2, particularly at pH 1.2 and 4.5, indicating the stabilizing effect of NaHCO_3_ on omeprazole release. At pH 1.2, OME release remained low in all the formulations, but degradation was evident in O1, while O2 exhibited slightly improved stability. At pH 4.5, O2 showed a higher percentage of released OME than O1, demonstrating enhanced drug protection.

A statistically significant difference was also observed when comparing O3 with O2 at all pH values except for the alkaline pH 6.8. The highest drug release at pH 6 was recorded for O3, followed by O2 and O1. At pH 4.5, O3 exhibited significantly higher drug release than O2. At pH 6.8, all the formulations showed comparable release levels, suggesting that the alkaline environment facilitates OME solubilization regardless of formulation type.

A relevant observation arising from the comparative evaluation of formulations O2 and O3 pertains to the differences in omeprazole release under various pH conditions. Both formulations, due to the disruption of the enteric coating during preparation, should be classified as immediate-release dosage forms rather than enteric-coated ones. It may be hypothesized that the mechanical fragmentation of the pellets in formulation O2 could result in partial retention of the drug within the excipient matrix, thereby contributing to the slightly lower release values observed. However, this interpretation requires careful consideration. As illustrated in Figure 1a, the enteric-coated commercial product had already demonstrated partial drug release at pH 6—corresponding to post-prandial gastric conditions—reaching approximately 50% of the value observed at pH 6.8. Notably, at pH 3 and 4.5, no drug release was observed from the intact pellets, suggesting that the release results for formulation O2 under these conditions may be underestimated. This finding indicates that the enteric coating may undergo partial dissolution in slightly acidic conditions, enabling earlier-than-expected drug release even in intact dosage forms. Consequently, the differences in drug concentrations between formulations O2 and O3 may reflect not only variability in the release process but also differences in the extent of degradation, which is not fully captured by static in vitro models. Moreover, it should be emphasized that the reproducibility of pellet crushing in pharmacy practice remains uncertain, and the present analysis was limited to a single commercial omeprazole product. Further investigations employing dynamic gastrointestinal models and a broader selection of commercially available preparations are warranted to validate and expand upon these findings.

## 4. Discussion

Despite significant progress in pharmacotherapy and the introduction of new active substances to the pharmaceutical market, there is still a lack of appropriate drug formulations, particularly those intended for pediatric and geriatric patients. Therefore, to adapt treatment to the needs and requirements of these patient groups, it is crucial to consider the personalized preparation of compounded formulations using appropriate pharmaceutical raw materials.

Hence, the present study aimed to assess the degradation rate of omeprazole depending on the pH of the environment and to propose recommendations for the preparation and administration of compounded omeprazole suspensions. These recommendations may help address some of the therapeutic challenges associated with this drug, particularly in pediatric patients and adults with dysphagia.

It is commonly observed that patients who, for various reasons, cannot swallow whole tablets or capsules tend to crush, split, or empty the capsule contents and mix them with food or beverages. However, this practice does not guarantee the stability of the active substance or ensure proper bioavailability. Any modification of the original drug form to adjust the dose for administration may introduce the risk of dosage errors, potentially affecting drug absorption and bioavailability. Enteric-coated tablets and capsules—including PPIs—should not be split or crushed, as doing so may destroy the active substance with a consequent lack of pharmacological effect.

Omeprazole irreversibly inhibits the final step of acid production at the secretory surface of the gastric mucosa [13,14] and effectively suppresses the H+/K+ ATPase enzyme only when acid secretion is actively occurring. Therefore, it is typically administered before meals to maximize the activation of proton pumps triggered by food intake [14,15]. However, the sodium bicarbonate in omeprazole suspension may influence proton pump activity independently of meals [16]. This suggests that administering immediate-release omeprazole at bedtime, regardless of food intake, may be both beneficial and convenient for many patients.

The available literature frequently emphasizes that the addition of sodium bicarbonate is crucial for enhancing omeprazole absorption in immediate-release formulations, as it protects uncoated omeprazole from acid degradation in the stomach. Studies have shown that when healthy volunteers received a 40 mg dose of uncoated omeprazole on an empty stomach (without sodium bicarbonate), the mean peak concentration (C_max_) and the area under the plasma concentration–time curve (AUC) were significantly lower. In contrast, co-administration of the same dose with 30 mmol of sodium bicarbonate resulted in substantially higher plasma concentrations and AUC values [17,18,19].

Furthermore, pharmacodynamic studies by Howden et al. [3] demonstrated that the antisecretory effect of immediate-release omeprazole occurred more rapidly than that of delayed-release omeprazole. Additionally, morning administration of immediate-release omeprazole provided effective postprandial acid control throughout the day, an effect that was evident from the first day of treatment and sustained over a week. According to Goldlust et al., adding a bedtime dose of immediate-release omeprazole maintained the gastric pH above 4 for a significantly longer duration than morning dosing alone, both overnight and over an entire 24 h period [20].

These observations led the authors of the present study to conduct innovative experimental research to explore different methods for preparing omeprazole suspension and compare the degradation rates of omeprazole in these formulations. Such studies can be highly relevant in clinical practice, as administering solid dosage forms poses a challenge for pediatric, geriatric, and adult patients with swallowing disorders (dysphagia). This necessitates the development of alternative drug delivery methods. One such approach is a pharmacy-prepared suspension with the composition described above. This formulation allows for precise dose adjustments tailored to individual patient needs, offering a high degree of personalized therapy. Moreover, it facilitates drug administration across all patient groups, particularly benefiting those with dysphagia.

This study demonstrated that the composition of the formulation significantly influences the omeprazole stability and release profiles. The results indicate the following:

1. The O1 formulation is not recommended due to its instability in acidic conditions, leading to significant omeprazole degradation before it reaches the absorption site, making it unsuitable for patient administration.
2. The O2 formulation, which includes sodium bicarbonate, demonstrates improved omeprazole stability by increasing the pH of the suspension. This buffering effect reduces omeprazole degradation both in the formulation and during administration compared to O1, allowing better control over drug stability.
3. The O3 formulation, which involves pure omeprazole, offers a significant advantage over O2. It demonstrates higher drug concentrations in the dissolution medium, suggesting that excipients in the commercial O2 formulation may contribute to degradation.

The results presented in this paper refer to standard procedures used in adult patients. The aim was to indicate the problem of drug stability and propose solutions for manufacturing technology. The continuation will be to shift the emphasis to the pediatric population, which, due to the large variability in the conditions within the GIT, is one of the greatest challenges in establishing standard procedures for testing release in vitro.

Our future directions include a comparison of the impact of two preparation methods on API stability and degradation: one where the active ingredient is first suspended in glycerin (85%) before adding sodium bicarbonate, as described in the literature, and our method where glycerin is added last. Moreover, we aim to reduce the volume of the dissolution medium and adjust the doses to better simulate drug administration in pediatric populations.

Furthermore, future studies should consider the application of dynamic two-stage and multicompartment in vitro dissolution models, which may better reflect the physiological environment and provide further insight into drug behavior under gastrointestinal conditions.

## 5. Conclusions

Based on the study findings, we recommend preparing the omeprazole suspension according to the composition of the O3 formulation (which includes pure omeprazole), as it results in higher drug concentrations in the dissolution medium. Additionally, patients should take the suspension immediately after a meal, as this minimizes omeprazole degradation and enhances bioavailability. The practical relevance of this research lies in the availability of omeprazole as a pharmaceutical raw material and the lack of immediate-release omeprazole formulations in the Polish market. Clinical indications confirm the need for such a formulation, highlighting the importance of further research and the implementation of personalized omeprazole suspensions in pharmaceutical practice.

## Figures and Tables

**Table 1:** Composition of the tested formulations.

Formulation	Omeprazole (Pure)	Omeprazole (Crushed Pellets)	Sodium Bicarbonate	Water	Glycerin
	[g]				
O1	-	1.3 (0.02 OME)	-	-	-
O2	-	13.0 (0.2 OME)	8.0	74.0	5.0
O3	0.2	-	8.0	86.8	5.0

O1—crushing the pellets from a commercially available drug; O2—crushing the pellets from a commercially available drug suspended in an 8% sodium bicarbonate solution; O3—pure omeprazole in an 8% sodium bicarbonate solution. 1.3 g of crushed pellets contains 0.02 g of OME.

**Table 2:** pH values of formulations O2 and O3 (mean ± SD, *n* = 3).

Formulation	pH (Mean ± SD)
O2	8.18 (±0.09)
O3	8.44 (±0.03)

## Data Availability

The data presented in this study are available on request from the corresponding author.

## Supplementary Materials

The following supporting information can be downloaded at: https://www.mdpi.com/article/10.3390/pharmaceutics17050594/s1, Supplementary Table S1. OME release profile of the commercial product (CP) and the O1, O2, and O3 formulations at pH = 3.0, pH = 4.5, pH = 6.0, pH = 1.2, and pH = 6.8 (% mean drug release); Supplementary Table S2. Dissolution profile comparisons for the difference factor (F1) and similarity factor (F2) of the commercial product and the O1, O2, and O3 formulations.

## Abbreviations

The following abbreviations are used in this manuscript:

PPI	Proton Pump Inhibitor
OME	Omeprazole
IR	Immediate-Release
AUC	Area Under the Curve
ATPase	Adenosine 5′-TriPhosphatase
HPLC	High-Performance Liquid Chromatography
SD	Standard Deviation
API	Active Pharmaceutical Ingredient
