# Supplementary material for: Formulation Approaches for Optimizing Omeprazole Stability in Oral Liquid Dosage Forms
Source: Pharmaceutics. 2025 May 1;17(5):594. doi: 10.3390/pharmaceutics17050594 (PMC12115015; doi:10.3390/pharmaceutics17050594)
Supplement: Supplementary file 1 [file pharmaceutics-17-00594-s001.zip › pharmaceutics-3606250-supplementary.pdf]

pharmaceutics

MDPI

Supplementary Table S1. OME release profile of the commercial product (CP) and the O1, O2, and O3 formulations at pH=3.0, pH=4.5, pH=6.0, pH=1.2, and pH=6.8 (% mean drug release).

	pH 3.0				pH 4.5				pH 6.0			
time-point [min]	CP (%)	O1 (%)	O2 (%)	O3 (%)	CP (%)	O1 (%)	O2 (%)	O3 (%)	CP (%)	O1 (%)	O2 (%)	O3 (%)
1	0.00	1.87	5.12	10.48	0.00	7.50	26.06	27.86	0.00	25.51	34.73	11.55
5	0.00	3.18	5.84	13.26	0.00	9.58	21.57	33.27	0.75	29.69	32.18	67.48
10	0.00	3.89	7.06	10.54	0.00	10.70	27.14	42.86	4.18	30.31	27.69	66.58
20	0.00	4.66	7.46	9.09	0.00	10.98	26.29	40.40	15.53	29.60	26.99	73.91
30	0.00	5.46	8.16	4.66	0.00	11.35	27.42	35.51	18.15	31.35	34.85	68.53
60	0.00	6.21	7.44	2.77	0.00	12.56	27.83	32.50	28.44	29.49	33.29	72.78
120	0.00	8.57	7.98	0.77	0.00	12.55	26.57	32.08	30.98	27.34	34.97	73.32

	pH 1.2				pH 6.8			
time-point [min]	CP (%)	O1 (%)	O2 (%)	O3 (%)	CP (%)	O1 (%)	O2 (%)	O3 (%)
1	0.00	12.36	15.51	11.23	0.00	57.52	71.52	67.58
5	0.00	9.75	15.01	7.94	2.29	54.77	71.99	69.01
10	0.00	9.33	12.58	6.77	7.49	56.45	71.60	65.29
20	0.00	8.14	10.94	6.74	48.92	56.53	70.49	64.61
30	0.00	7.69	9.85	6.90	59.21	54.24	72.09	64.52
60	0.00	6.71	8.73	7.21	62.86	53.78	69.44	63.99
120	0.00	3.02	8.50	6.71	64.11	56.78	69.34	64.95

Supplementary Table S2. Dissolution profile comparisons for the difference factor (F1) and similarity factor (F2) of the commercial product and the O1, O2, and O3 formulations

	O1		O2		O3	
	F1	F2	F1	F2	F1	F2
pH 3.0	∞	63.66	∞	57.28	∞	53.32
pH 4.5	∞	48.11	∞	29.08	∞	22.64
pH 6.0	114.85	35.90	129.26	33.49	342.98	14.63
pH 1.3	∞	53.21	∞	46.18	∞	55.25
pH 6.8	76.76	22.66	102.76	16.88	87.84	18.58

F1: difference factor; F2: similarity factor; No. of time points: 7; reference: commercial product; ∞—infinity
